# Advanced technologies in plant factories: exploring current and future economic and environmental benefits in urban horticulture

**DOI:** 10.1093/hr/uhaf024

**Published:** 2025-01-27

**Authors:** Xin Yuan, Jiangtao Hu, Leo F M Marcelis, Ep Heuvelink, Jie Peng, Xiao Yang, Qichang Yang

**Affiliations:** Institute of Urban Agriculture, Chinese Academy of Agricultural Sciences, Chengdu National Agricultural Science & Technology Center, No. 36 Lazi East Rd, Chengdu 610213, China; Horticulture and Product Physiology Group, Department of Plant Sciences, Wageningen University & Research, Droevendaalsesteeg 1, Wageningen 6708 PB, The Netherlands; Institute of Urban Agriculture, Chinese Academy of Agricultural Sciences, Chengdu National Agricultural Science & Technology Center, No. 36 Lazi East Rd, Chengdu 610213, China; Horticulture and Product Physiology Group, Department of Plant Sciences, Wageningen University & Research, Droevendaalsesteeg 1, Wageningen 6708 PB, The Netherlands; Horticulture and Product Physiology Group, Department of Plant Sciences, Wageningen University & Research, Droevendaalsesteeg 1, Wageningen 6708 PB, The Netherlands; Institute of Urban Agriculture, Chinese Academy of Agricultural Sciences, Chengdu National Agricultural Science & Technology Center, No. 36 Lazi East Rd, Chengdu 610213, China; Institute of Urban Agriculture, Chinese Academy of Agricultural Sciences, Chengdu National Agricultural Science & Technology Center, No. 36 Lazi East Rd, Chengdu 610213, China; Institute of Urban Agriculture, Chinese Academy of Agricultural Sciences, Chengdu National Agricultural Science & Technology Center, No. 36 Lazi East Rd, Chengdu 610213, China

## Abstract

Plant factories (PFs), also known as vertical farms, are advanced agricultural production systems that operate independently of geographical and environmental conditions. They utilize artificial light and controlled environments to produce horticultural plants year-round. This approach offers a promising solution for the stable and efficient supply of high-quality horticultural produce in urban areas, enhancing resilient urban food systems. This review explores the economic and environmental impacts and potential of PFs. Breakthroughs in PF research and development are highlighted, including increased product yields and quality, reduced energy input and CO_2_ emissions through optimized growing conditions and automation systems, transitioning to clean energy, improved resource use efficiency, and reduced food transport distances. Moreover, innovations and applications of PFs have been proposed to address challenges from both economic and environmental perspectives. The proposed development of PF technologies for economic and environmental benefits represents a comprehensive and promising approach to urban horticulture, significantly enhancing the impact and benefits of fundamental research and industrial applications.

## Introduction

**Figure 1:**
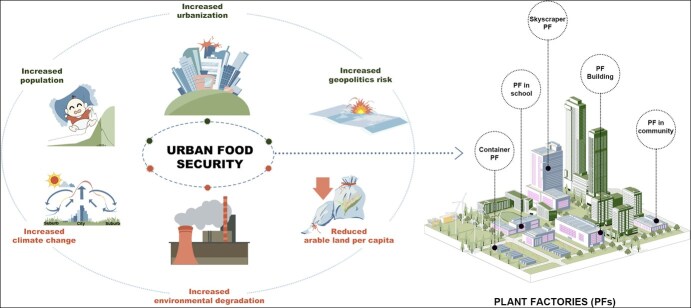
Plant factories as a promising solution to address the challenges of food security in urban areas. Note: Increased population, urbanization, geopolitics risks, climate change, environmental degradation, and decreased arable land per capita are believed to be the most harmful problems related to food security in urban areas. Plant factories are promising solutions, for example, container PF, PF in school, skyscraper PF, PF building, and PF in the community

With the rapid increase in global population and urbanization, it is projected that 67% of the world's population will reside in urban areas by 2050, and the total global population will reach 9.7 billion [1]. Urban horticulture is widely recognized for its significant role in the urban food system, aiming to sustainably feed approximately 2.2 billion new urban dwellers over the next three decades (Fig. 1) [1, 2]. Additionally, ongoing global urban expansion is expected to lead to a loss of 30 Mha of global cropland by 2030, equating to a 2% decline in global cropland area, resulting in a 3.7% reduction in global crop production, and thereby posing a significant global threat to cropland availability and food security [3]. Moreover, continuous environmental degradation (such as soil, water, and air pollution), climate change (e.g. urban heat island effects), and geopolitical events have extensive and profound implications for horticultural production in urban areas [4]. Consequently, urban horticultural production has highlighted a burgeoning trend towards high-yield, high-quality, efficient, environmentally friendly, and sustainable cultivation. There is a growing need for novel horticultural systems that ensure the efficient and stable production of safe and nutritious crops while supporting sustainable urban growth and complementing conventional farming methods such as open fields and greenhouses [5–7].

**Figure 2:**
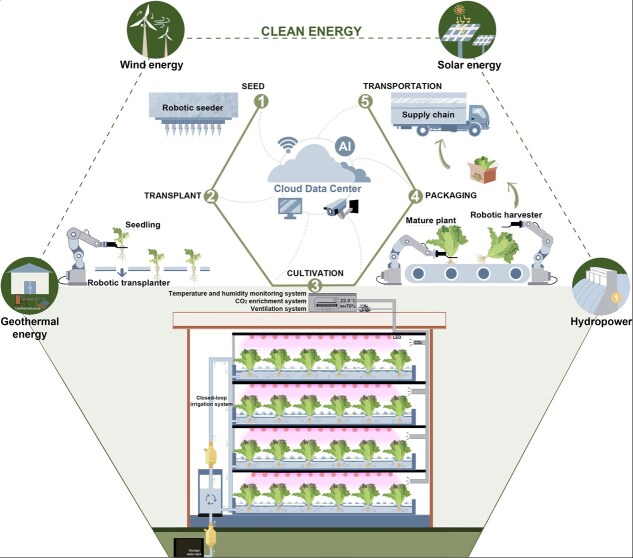
Schematic drawings of a brief production process for an automated PF with details on clean energy use, environmental control, and cultivation system. Note: Outside the PF envelope, there are production systems of clean energy, including ground-source heat pumps, wind turbines, solar panels, and hydropower plants. Within the automated PF, the producing line consists of robots of seeder, transplanter, harvester, packaging system, and cultivation systems. Multilayer tanks, lighting systems, air temperature and humidity monitoring, CO_2_ enrichment, ventilation, and close-loop irrigation are used within the cultivation system. Moreover, all devices will be controlled and feedback data, forming the automatic loop with the help of the IoTs, sensors, artificial intelligence, and the cloud data center

Plant factories (PFs), also known as vertical farming systems, are advanced, enclosed agricultural systems that enable the vertical cultivation of horticultural plants in multiple layers with precisely controlled environmental conditions and fertigation (Fig. 2). PFs represent a cutting-edge horticultural production system that may integrate hydroponic cultivation, horticultural robots, database management, the Internet of Things (IoTs), automation, artificial intelligence (AI), and green architecture technology. They are considered a potential approach to meet the demands of year-round production of horticultural products in a high-yield, high-quality, effective, green, and sustainable manner. Compared with conventional horticultural production methods such as greenhouse and open-field cultivation, PFs are much less land-intensive, require less water, nutrients, and pesticides, and are not constrained by geographic and environmental factors. Consequently, PFs can effectively supplement limited cropland and urban open space and utilize unused urban spaces. Over the last decade, PFs have emerged as a significant investment sector, with numerous commercial PFs established in East Asia, Europe, and North America, including China, Japan, South Korea, Singapore, the Netherlands, Germany, France, the United Kingdom, Sweden, Denmark, the United States, and Canada [8]. The global market size of PFs was valued at 8 billion USD in 2024 and is projected to reach 35.3 billion USD by 2032 [9]. Furthermore, consumer demand for PF products is gradually increasing due to the growing emphasis on food safety, high quality, and consistent supply [10]. Nevertheless, owing to the high construction and operational costs of PFs, high energy use, and necessity for sustainable practices in urban areas, there is growing interest in cutting-edge research and development, with a focus on PFs with economic and environmental benefits.

In this review, the current and potential benefits of PFs are highlighted from the perspectives of diverse crop production and quality as well as the global perspectives of the highly efficient resources use, carbon footprint reduction, and the recycling and reuse of waste. Moreover, this review proposes potential areas for further research that could contribute to the economic and environmental benefits of PFs, providing a basis for their expanded application in urban horticulture.

## Advanced technologies contributing to the economic benefits of PFs

The economic benefits of PFs are critical for industrial applications, including land use and construction costs, energy consumption, labor, agricultural supplies, harvesting, and postharvest storage [11–13]. The selection of highly profitable plant species and the application of cutting-edge technologies for precise environmental control are essential for enhancing the economic viability of PFs by improving product yield and quality.

### Plant species cultivated in PFs with high profits

The production of nutritious and healthy horticultural products in PFs is a promising avenue, with the potential for an industrial boom with considerable economic benefits. The most consumed horticultural crops grown in PFs are microgreens and leafy vegetables. These plants have short life cycles, relatively high prices, and significant market demand. Microgreens (e.g. pak choi and radishes with two true leaves) and leafy greens (e.g. lettuce and basil) are particularly popular owing to their common utilization in salads, rapid growth (harvestable 10–40 days after sowing or transplanting), compact size (enabling a high plant density and multilayer cultivation), high yield (harvest-index >85%, indicating that more than 85% of the plant biomass is edible and available for sale), and high quality (e.g. high levels of health-promoting compounds) [14]. These factors contribute to premium net profit margins. In addition, to enhance the profitability of PFs, expanding the cultivation of highly profitable horticultural species, such as cannabis and transplants can be advantageous [15, 16]. There is also an emerging market for flowers, ornamental plants, fruits (e.g. strawberries and melons), solanaceous plants (e.g. pepper and tomato), and medicinal plants [16–18]. Moreover, the recent expansion of value-added agriculture has provided significant opportunities to boost the PF industry. There are opportunities for operation diversification, including applications in biopharmaceuticals (see section 4.7), and branding. PFs equipped with precise environmental control and fertigation systems can manipulate the composition and concentration of health-promoting compounds in horticultural plants, thereby facilitating the production of biofortified horticultural plants, particularly those with health benefits, such as antioxidant activity, anti-aging properties, and the preventive effects against chronic diseases [19–21].

### Improvement of product quantity and quality by environmental optimization contributes to market value

Numerous environmental factors influence the yield and quality of horticultural plants, including their sensory characteristics and nutrient composition, which determine market value. Here, individually or in combination, environmental factors that contribute to photosynthetic efficiency, morphological adaptations, and antioxidant accumulation, are reviewed in detail. These factors include light, temperature, air humidity, CO_2_ concentration, nutrition, and airflow.

#### Effects of light on plant yield and quality

Light is a crucial factor in the growth and development of horticultural plants, with artificial lighting accounting for 23%–27% of the total operating costs in a PF [16, 22]. Therefore, optimizing artificial lighting systems is vital for efficient horticultural plant production in PFs. The total output or mass of horticultural plants is often related to the total amount of radiation absorbed, which depends primarily on light intensity and photoperiod. Light-emitting diodes (LEDs) are the preferred light source for horticultural plant cultivation in PFs because they are efficient, easily controlled, adjustable, and emit cool light [23–25]. Since all light in PFs comes from artificial lighting, light input is quantified as daily light integral (DLI; mol m^−2^ d^−1^), defined as light intensity multiplied by photoperiod. Biomass accumulation of herbaceous plants increases by approximately 1% for every 1% increment in DLI from 5 to 25 mol m^−2^ d^−1^ across growth chambers, greenhouses, and open field conditions [26, 27]. Therefore, enhancing the overall amount of radiation a horticultural plant receives daily may be a practical strategy for enhancing the biomass accumulation of leafy greens in PFs [28, 29]. Such an outcome can be achieved by adjusting the light intensity and photoperiod combination based on the species and cultivars.

In addition to light intensity and duration, light quality has significant effects on plant growth and development in PFs, particularly on phytochemical accumulation in horticultural plants [30]. Within the range of photosynthetically active radiation (PAR; 400–700 nm), a large fraction of red light (600–700 nm) induces increases in biomass, leaf area, leaf length, height, and soluble sugar content, and decreases in carotenoids and chlorophyll in leafy vegetables [24]. In contrast, a large fraction of blue light (400–500 nm) suppresses plant growth including leaf area development and height but promotes the accumulation of phytochemicals, such as chlorophyll, carotenoids, total phenolics, anthocyanin, and glucosinolate [24]. The increase in anthocyanin may be due to the upregulated expression of the biosynthesis genes chalcone synthase and chalcone isomerase [31]*.* A red to blue light ratio (R:B = 3) stimulates plant growth and increases overall biomass within the range of 0.5–4 [32, 33]. A high proportion of red light (>50%) in a mixed red and blue light spectrum increases plant monoterpenoid and sesquiterpenoid contents but inhibits the synthesis of plant tetraterpenoids, which are mainly regulated by light signal transduction transcription factor elongated hypocotyl 5 (HY5) [34]. The balance between growth enhancement and phytochemical enrichment is crucial for optimal plant cultivation outcomes. Green light is on average as effective in increasing plant dry mass as a mixture of red and blue light [35]. Nevertheless, green light slightly increased plant fresh weight, associated with larger leaf area, thinner leaves, improved intrinsic water use efficiency, and decreased stomatal conductance [35].

In addition to the PAR range, the effects of ultraviolet B light (UV-B; 280–315 nm), ultraviolet A light (UV-A; 315–400 nm), and far-red light (FR; 700–800 nm) on yield and quality have been examined in lettuce, microgreens, and other horticultural crops [36–38]. UV-B light influences lettuce growth by inhibiting hypocotyl growth, expanding cotyledons, and enhancing flavonoid synthesis [39]. However, excessive UV-B levels can adversely impact plant health by causing DNA damage, inducing reactive oxygen species production, and disrupting photosynthetic processes [39]. Supplementing white light with 10 μmol m^−2^ s^−1^ UV-A light (370–390 nm) resulted in decreased leaf size, reduced lettuce biomass, and lower nitrate levels [40]; conversely, it increased the levels of chlorophyll, soluble protein, soluble sugar, vitamin C, flavonoids, polyphenols, and anthocyanins and enhanced the radical-scavenging capacity [40]. UV-A1 light (358–383 nm) had minor effects on the morphology of tomato, lettuce, and cucumber but did not affect biomass, regardless of the PAR background range [41]. FR can increase the shoot: root ratio in tomato, which is modulated by phytochromes B1/B2 [42], and stimulates dry weight partitioning to tomatoes by increasing the sink strength of fruits [43]. Furthermore, the effects of FR on leaf area in lettuce may depend on total light intensity, which can be attributed to differences in biomass partitioning between leaves and stems [44]. However, in cucumber, FR stimulated an increase in leaf area, independent of total light intensity [44]. Adding FR to white light (11%) increased the rate of photosynthesis and daily carbon uptake in individual leaves across the entire tomato canopy [45]. The effects of light quality on plant quality and quantity vary among plant species and growth stages. Therefore, optimizing light recipes for specific horticultural crops is important, to ensure a high yield and consumer acceptance of beneficial phytochemicals in commercial PFs.

Top lighting is widely used in the production of horticultural plants in PFs. Maintaining a consistent total light intensity while modifying the direction of artificial light exposure is a potential strategy for increasing the yield and quality of horticultural crops. The partial replacement of top lighting with side lighting has been reported to promote plant growth and runner formation in strawberry cuttings and accelerate flowering and additional branch formation in chrysanthemum cuttings [46, 47]. Moreover, upward lighting, directed from below the plant canopy, provides supplemental light to the lower leaves, thereby increasing the photosynthetic rates of the entire plant. These beneficial effects have been documented in studies of komatsuna and roses [48, 49]. Drawing inspiration from interlighting techniques used in high-wire tomato cultivation in greenhouses [50], methods for modifying the direction of artificial lighting hold significant potential for widespread application in PFs.

#### Effects of temperature on plant yield and quality

The annual electricity consumption for cooling in PFs accounts for 15%–35% of the total energy usage, making it the second-highest energy cost following lighting [16]. For instance, the fresh yield of lettuce is highest at an air temperature of 24°C when light intensities range from 200 to 750 μmol m^−2^ s^−1^ [51]. In analyses of the effects of short-term preharvest management on the yield and quality of lettuce, Zhang *et al.* found that quality indicators, including contents of soluble solids, most phenolic compounds, fructose, and glucose, were higher at 10°C than at 22°C, without yield loss [52]. Moreover, treating plants with day temperatures higher than night temperatures (positive DIF) compared with the reverse (negative DIF) results in increased shoot and leaf weight, leaf length, number of leaves, leaf area, and height in lettuce and basil [53]. Future research should focus on breeding or screening crop varieties suitable for cultivation under a wide range of temperatures, thereby reducing the energy consumption required for temperature control.

#### Effects of air humidity on plant yield and quality

Air humidity, as described by relative humidity (RH) or vapor pressure deficit (VPD), is one of the most critical factors affecting plant transpiration in PFs [54]. The optimal relative humidity range can enhance yield by modulating stomatal conductance, plant transpiration, photosynthesis, and nutrient absorption. Carbon gain and dry matter accumulation in lettuce are higher under a low VPD (<0.7 kPa; high RH) than a high VPD (>1.8 kPa) via improved stomatal opening, and similar effects have been observed in kale and tomato seedlings [55, 56]. Conversely, high VPD, as a mild stressor, contributes to synthesizing antioxidants, such as total ascorbic acid and phenol in lettuce [57], and increases hydrophilic antioxidant glucosinolate content in kale [55]. Excessively high RH (low VPD) can lead to a reduced leaf transpiration rate and decreased root absorption of nutrients such as Ca^2+^, potentially resulting in tip burn in lettuce, whereas low RH decreases stomatal conductance, increases transpiration, and may ultimately lead to plant dehydration [58].

#### Effects of CO_2_ on plant yield and quality

Enrichment of CO_2_ improves photosynthetic assimilation and the nutritional value of plants. Elevated CO_2_ concentration (generally 800 μmol mol^−1^ compared with the atmospheric level of 400 μmol mol^−1^) increases fresh weight and concentrations of antioxidants, ascorbic acid, and glutathione in broccoli and basil by enhancing leaf photosynthetic rates [59, 60]. As one of the most important factors in promoting the growth of horticultural plants, CO_2_ is commonly added in controlled environment agriculture facilities, such as greenhouses and PFs, with the potential to increase yield and productivity by 9%–45% [61].

#### Effects of nutrition on plant yield and quality

Electrical conductivity, pH, and dissolved oxygen concentrations of nutrient solutions are critical in optimizing the root zone environment, plant growth, and health [62]. For example, electrical conductivity requirements vary significantly among plant species and growth stages; 2–2.5 dS m^−1^ is suitable for tomato growth, whereas half of that range is suitable for leafy vegetables [63]. Maintaining high dissolved oxygen levels is crucial for promoting yield in the deep-flow technique, which could explain the increase in shoot biomass of lettuce under flow conditions compared to that in nonflow root environments [64]. Recent research suggests that exogenous glycine (molecular organic nitrogen) in hydroponic systems can enhance the accumulation of glycosylated flavonoids in lettuce [65, 66]. Nutrient solutions, such as selenium, zinc, and iodine, contribute to plant biofortification [19–21]. Hu *et al.* [20] proposed a production system for precisely controlling selenium (Se) content based on PF technology to regulate total Se and seleno-amino acid contents. Controlling daily Se intake is vital to avoid health risks associated with intake levels that are lower or higher than the recommended ~60 μg per day [67]. In addition, adding microbial inoculants (Arthrobacter pascens BUAYN-122 and Bacillus subtilis BUABN-01) to the hydroponic system can enhance both the quantity and quality of lettuce and celery. This enhancement is achieved by boosting root nutrient uptake and leaf photosynthesis, resulting in higher shoot fresh weight, total protein, vitamin C, total phenol, anthocyanin, and flavonoid contents [68]. An appropriate fertilization strategy is essential to produce premium products with optimized economic benefits by balancing plant yield and quality.

#### Effects of airflow on plant growth

Airflow is a critical factor in plant growth in PFs because it shapes the microclimate around the plants and directly affects photosynthesis and transpiration rates. Tip burn is a common issue in several crops cultivated in PFs, particularly under low airflow conditions, and is often attributed to a calcium deficiency (e.g. in lettuce) [69]. Maintaining a constant airflow above 0.28 m s^−1^ can significantly reduce the incidence of tip burn by enhancing transpiration and calcium delivery to the leaf tips [70]. In a ventilation system simulated by a computational fluid dynamics (CFD) model (which simulates fluid flows using algorithms and fluid mechanics), the diameters and number of pores have a combined impact on the airflow pattern [71]. In addition, airflow in PFs is primarily influenced by air inlets rather than outlets, and local environmental controls, such as perforated air tubes at each cultivation layer, contribute to a uniform microclimate around lettuce, as demonstrated by CFD simulations [72]. Moreover, exposure to strong winds (2–4.7 m s^−1^) induces mechanical stress on plants, resulting in shorter tomato seedlings with thicker stems and leaves, providing an effective strategy to manage compact plants [73]. Further research is required to determine the molecular mechanisms underlying the effects of different airflow rates on plant growth in PFs.

### Optimizing environmental conditions to promote the growth and quality of horticultural plants for maximum benefits

In horticultural practice, optimizing multiple environmental factors to achieve a balance between yield and quality is a key to maximizing economic benefits. Selecting a specific light spectrum that reduces elongation and a negative DIF might result in a compact plant architecture. The combined effects of light intensity, air, and root temperature on lettuce growth have been evaluated [51]. A multiple-factor energy-yield model has also been applied to the PFs, considering factors such as light intensity and periods, VPD, CO_2_, and air temperature [74]. In the future, multi-objective optimization models for environmental control can be implemented in production to increase economic benefits.

## Cutting-edge technologies increase environmental benefits in PFs

The PFs have been proposed as viable and sustainable food production systems; they have the potential to enhance the resilience of the local food supply in urban areas [75–77]. The use of clean energy, increasing energy use efficiency, reusing output waste, reducing food transport distances, and integrating intelligent control aim at promoting environmental sustainability.

### Clean energy transition for sustainable development in PFs

Owing to the extensive utilization of energy for maintaining optimal conditions for horticultural plant growth and development, PFs contribute to the carbon footprint in agriculture [78]. Fossil fuel is the primary source of generating electricity for PFs [79], which has detrimental effects on ecosystems. With the rapid development of clean energy technology and the significant decline in clean energy costs in recent years, PFs can integrate clean energy sources to reduce environmental impacts [80–83]. Solar photovoltaic, wind and hydro energy are reshaping the global electricity supply [84]. An increased proportion of hydro and wind energy reduced CO_2_ emissions by approximately 23% in life cycle assessment in Sweden, from 0.98 (an electricity mix of 41% nuclear, 39% hydro, 10% wind, 9% combined heat and power, and 1% solar) to 0.75 (50% hydro and 50% wind) kg CO_2_ equivalent kg^−1^ [82]. Moreover, the ground-source heat pump can decrease greenhouse temperatures in the summer using cold groundwater and vice versa [85]. Clean energy is considered a viable resource for the PF industry, despite various challenges, such as the unstable power supply and relatively low energy conversion efficiency. It is projected that by 2050, 70% of global electricity will be generated by renewable power to achieve worldwide net-zero emissions [86]. In addition to renewable energy, the share of global nuclear energy power production (type of clean energy) is expected to increase from 9% in 2023 to 13% by 2050 [87]. The projected scaling up of the energy industry could address the unstable power supply and enhance energy conversion and land use efficiencies in the coming years.

Another energy transition technology that could be applied in PFs is clean energy storage (e.g. storing electricity in batteries) for efficient use and flexibility [88]. When integrated with electricity production, energy storage can act as a conduit for energy transfer within an optimal energy-scheduling framework to maximize energy utilization efficiency. The storage system can regulate the balance of the mismatch between electricity production and the grid, storing excess energy and using it for power shortages [89].

### Green technologies to meet the growing demand for sustainability

PFs consume a considerable amount of electricity, whose cost accounts for 25%–30% of the total operating cost [16, 90]. Previous studies have reported that theoretical and experimental electricity used for a PF to produce lettuce ranges in 25–210 g (fresh weight) kWh^−1^ [80–82, 89, 91–94]. Reducing the environmental impact is a key goal in the PF industry. In this regard, energy-saving technologies are important for innovation and transfer in the PF industry or its sustainable development [92]. For instance, increasing the conversion efficiency of electric energy to photons in LEDs could reduce electricity consumption and carbon emissions significantly, while maintaining the same DLI for plant growth [25, 95]. Also, developing an appropriate model for simulating indoor thermal environments is crucial for advancing energy-efficient and innovative technologies [96]. For instance, optimizing energy consumption in PFs involves clarifying the energy flow and balance in the operation and applying yield-energy models to minimize energy consumption [93]. Further investigations are required to employ an integrated approach incorporating advancements in building materials, heating, ventilation, air conditioning, and lighting, as well as generation technology to reduce electricity consumption in PFs.

As a closed agricultural production system, PFs can maximize the utilization efficiency of natural resources, which is conducive to the sustainable development of agriculture. For example, according to a model-based study, lettuce production in PFs with regain of transpired water can reduce the total water use by up to 95% compared to common greenhouses in the Netherlands [95]. A scalable hygroscopic gel could help to regain water from passive plant transpiration [97]. In addition, CO_2_ and inorganic fertilizers (e.g. nitrogen, phosphorus, and potassium) were utilized with efficiencies of 87%–89% and 85%, respectively [95, 98]. Using CO_2_ capture and storage technology to fix high concentrations of CO_2_ in PFs for plant photosynthesis can further improve the use efficiency of CO_2_ and reduce CO_2_ emissions by balancing the generation and consumption of CO_2_ during the day and night [61]. However, energy consumed by recycling water, fertilizers, or CO_2_ cannot be neglected under current technological conditions.

### Waste output reuse technologies for ecological benefits

Waste reuse technology is important for the sustainability of PFs. Waste outputs from horticultural plant production include crop residues (e.g. roots and stems), waste heat energy, and used substrates [99, 100]. As approximately half of the electric energy input into PFs is converted into waste heat energy, it is important to reuse it to reduce CO_2_ emissions [14, 101]. In addition, the waste heat from PFs can be used for space and water heating in buildings [84], and local heat networks reduce energy use by up to 15% compared with natural gas [102], realizing synergetic benefits for urban communities. Moreover, the main component of crop residue, cellulose, can be a sustainable source of biofuel and bioenergy [103]. For example, superheated steam torrefaction can efficiently convert urban agricultural waste into high-calorific value fuels [104]. The reuse of growing medium and lettuce roots in a mushroom substrate has been evaluated in a commercial PF [82]. Reusing waste fertilizers and plastic products generated by PFs is also recommended to promote sustainability [14, 101]. While the current high costs associated with waste reuse highlight the need for technological advancements.

### Fewer food miles in PFs for sustainability

Total food miles correspond to approximately 19% of the global food system emissions, 36% of which can be contributed to vegetables and fruits [105]. Given that most food transportation occurs between production areas and urban centers, developing PFs within cities has the potential to reduce greenhouse gas emissions substantially by minimizing food miles [106]. It was estimated that the average food miles for PFs (e.g. 43 km) are significantly lower than those for greenhouses and open fields (800–1600 and 3200 km, respectively) [107]. Among various modes of transportation, roads accounted for the majority of the emissions (81%), followed by trains, ships, and airplanes [108].

### Internet of Things system for improving energy use efficiency and productivity

The Internet of Things (IoT), embedded with sensing, decision-making, and operational processes, is a component of PFs [109, 110]. The rapid advancement of IoT devices, including sensors, communication technologies, and data processing units, has enabled the integration and application of IoT in PFs [111, 112]. Moreover, IoT-based accessory machinery (e.g. seeders, transplanters, and harvesters) have been widely applied in PFs [113]. Thus, IoT-based control systems have been applied in commercial PFs (e.g. household, container, and building types) and incorporate a multitude of functions (e.g. seed plot scheduling, smart production, agricultural input, environmental and fertigation control, and energy consumption control) [5, 114, 115]. The integration of IoT enhances sustainability by increasing crop productivity, operational efficiency, and nutrient use efficiency, in addition to reducing energy and labor in PFs.

## Perspectives

Urban agriculture is a promising solution for the challenges posed by growing populations and decreasing availability of agricultural land. PFs have made noteworthy progress in demonstrating their economic and environmental benefits [84]. PFs play an important role in solving urban horticultural issues such as economic viability and environmental sustainability. However, the development of PFs remains challenging because of high energy use, construction, and operation costs. Therefore, numerous factors should be considered to facilitate the broader application and continued innovation of PFs.

### A unified unit for energy assessment in PF comparability

Previous studies have demonstrated that electricity costs for producing leafy vegetables in PFs vary considerably, reported in different units of measurement (e.g. kg kWh^−1^, kWh kg^−1^, and kWh m^−2^ y^−1^) [80–82, 91–95, 116]. The conversion from impact per square meter or year to impact per kWh is not straightforward, as multiple processes may be involved in production. Economic costs are calculated or predicted based on several factors, including production areas, vertical layers, planting density, cultivation cycles per year, total light integral, and other environmental conditions, which vary for different PFs. Therefore, it is necessary to establish a unified unit to calculate the economic cost of a PF. It is proposed that g(fresh weight) kWh^−1^, the unit of fresh products produced by one kWh of electricity and an indicator of energy use efficiency, should be adopted as a reliable and convenient basis for comparison. In addition, because industrial electricity prices vary widely from country to country, production costs in € kg^−1^ should also be reported as supplementary information. Here, the selling prices of lettuce in commercial PFs across multiple countries were provided as an example (Supplementary Table S1).

### Breeding specialized crops for PFs

The diversity of crops produced in PFs is currently limited. To improve economic and environmental benefits, PFs favor plants with a rapid growth cycle, compact stature, high quality, high yield, functionality, tolerance to a low light intensity, a high edible fraction, high-use efficiency of nutrients and light, and ease of robotic handling, ultimately achieving quick and continuous production [117, 118]. Recent breakthroughs highlight the potential for breeding specialized cultivars tailored to PF environments. For instance, Zhang *et al.* [119] developed a new lettuce cultivar, 'Zhongsheng No.1′, bred for cultivation in PFs.

Genome editing provides a method for designing traits suitable for PF cultivation [120]. Several countries are developing commercially available genetically modified products [121–123]. For example, CRISPR–Cas9 technology has been used to transform the vine-like tomato genotypes into compact, early-yielding forms without yield penalties. These modifications result in plants suitable for high planting densities and multilayer cultivation in PFs, thereby maximizing annual production [124]. Similarly, genome editing facilitates the mechanical harvesting of tomato fruits without flavor loss or physical damage [125]. This approach can be applied to increase the proportion of edible parts (fruits, leaves, stems, and roots) while enhancing nutrient contents and achieving higher yields [126].

### AI-based technologies in the next generation of PFs

Artificial intelligence (AI) technology has advanced rapidly and is now widely adopted in various aspects of daily life. However, its application in agriculture, particularly high-tech PFs, remains limited [127]. Recent research suggests that AI could be used to enhance the breeding efficiency of horticultural plants and, in collaboration with the IoTs could improve operational performance, thereby optimizing economic and environmental outcomes. Integrating AI and genome-editing technologies in PFs allows for the precise design of horticultural plants tailored to specific growth conditions. For example, the CropGPT project focused on AI-driven breeding programs based on biological big data, aims to develop new varieties with optimized productivity, quality, and adaptability to cultivation constraints, thereby increasing profitability and exploiting the potential of PFs [121]. Moreover, to optimize crop growth conditions and maximize yields, dense networks of wireless sensors (e.g. environmental sensors for light, air speed, and humidity, as well as physiological sensors for plant color and stem diameter) can be utilized. These sensor networks, collectively known as the Internet of Plants, can be integrated with AI [128]. The ultimate artificial intelligence-of-things (AIoT) model involves real-time data acquisition through the Internet of Plants and Things, analysis, decision-making, and breeding. This model can facilitate intelligent plant growth monitoring in the next generation of PFs, thereby enhancing sustainability by increasing crop productivity, resource use efficiency, energy savings, and labor demand requirements [129].

### Roles of multiple parties in boosting PFs

The rapid growth of the PF industry is limited by high construction and operation costs, which could be addressed through collaborative efforts involving multiple parties. Governments play a critical role in the innovation and application chain, acting as a catalyst for PF development. Governments should establish cross-sectoral and cross-domain coordination and decision-making mechanisms, fostering a governance environment that involves the efforts of government, market, and society. They should develop infrastructure, implement priority planning regulations, and offer financial investments or supplementary allowances to lower the economic barriers associated with PF adoption. For example, the Chinese government has encouraged multiple parties involved in the expansion of the PFs industry in urban areas recently [130]. Expanding PFs and reducing their construction and operation costs requires investment from governments, venture capitalists, and related stakeholders in research initiatives that focus on developing and applying advanced technologies.

### PF-based speed-breeding technology accelerating horticultural plant research and breeding

To date, PF-based speed breeding is increasingly utilized to accelerate breeding programs. The cultivation of horticultural plant materials and their parent plants in a PF environment can shorten the growth cycle by providing an appropriate environment for flowering and seeding, facilitating the development of new cultivars [131, 132]. This approach enables breeders to produce new generations continuously year-round, regardless of environmental or geographical conditions [133]. For instance, Zhang *et al.* [119] reported that PF technology significantly shortened the duration of a single life cycle in PF to 90 d compared with 180 d under open-field conditions. Lettuce lines were then crossed with other lines, and hybrid offspring were selected from PF-based breeding and open fields, reducing the breeding cycle by 50%. Furthermore, approving a new horticultural plant cultivar requires an evaluation of its environmental adaptability through a cultivation experiment in areas previously identified as suitable. PFs could be used to simulate different environmental conditions and to assess the relationships between environmental factors and horticultural plant growth and metabolic dynamics, which offers a convenient approach for performing cultivation experiments independent of geographical conditions [134]. Moreover, integrating PFs with other breeding technologies could shorten breeding times considerably. For instance, combining modern breeding techniques, such as genetic engineering and marker-assisted selection, with PF-based speed breeding technology allows for the efficient selection of elite genotypes with novel features [135].

### PFs lighting up urban life

PFs light up urban life by providing spaces for education and leisure and offering opportunities for engagement and experiential learning. Efforts to popularize PFs through educational initiatives (e.g. extracurricular activities for teenagers) could improve public awareness and acceptance. As part of urban agriculture, community and household PFs offer residents opportunities to cultivate and harvest horticultural plants [2, 5, 136]. Moreover, PFs can be constructed alongside businesses, including shopping malls, restaurants, and hotels, allowing these establishments to offer fresh products onsite. These activities would increase awareness of the economic and environmental benefits of PFs and encourage active community engagement in urban agriculture, contributing to green decoration, leisure, and sustainability.

### Safe and efficient production of bio-based products in PFs

Plant synthetic biology has advanced the design and construction of complex biological systems through engineering, enabling the development of numerous products that have rapidly permeated society [137]. PF-based production systems have been proposed as viable alternative sources for several synthetic biology-based products, including genetically modified organisms (horticultural plants), natural products (plant secondary metabolites), and vaccines [138]. For instance, Zhang *et al.* [139] developed a melatonin-enriched tomato cultivar by engineering light-induced melatonin biosynthesis; this cultivar could be produced in PFs and exert beneficial effects on the human circadian rhythm and sleep. Moreover, PFs have been proposed for the automated, standardized, and efficient production of various biopharmaceuticals in a cost-effective, scalable, and safe manner. These products include virus-like particles, miraculin in dwarf tomatoes, functional proteins in strawberry, cannabinoids in cannabis, and artemisinin (also 'Qinghaosu') in sweet wormwood [140–144].

### Green building technologies enhancing the suitability performance of PFs

The agricultural sector is a major consumer of nonrenewable energy and a prominent source of greenhouse gas emissions. Given the high energy demands of PFs, there is a clear need to reduce CO_2_ emissions associated with their construction and operation. The CO_2_ emissions associated with PF buildings are often overlooked. Prefabricated buildings can result in significantly lower emissions than those associated with conventional construction methods during the materialization stage [145]. Furthermore, carbon-neutral buildings can be constructed in PFs [146]. Relevant technologies include the utilization of renewable resources and eco-friendly materials to minimize the environmental impact of PFs, the incorporation of advanced thermal materials in external and roof walls to reduce heat transfer coefficients, and the installation of amorphous silicon solar photovoltaic tiles or solar panels to generate renewable electricity and heat energy [136]. The development of PFs may increasingly focus on green and low-carbon architectures (such as containers and buildings). Moreover, efficient cultivation management can reduce greenhouse gas emissions significantly. PFs have the potential to serve as models for green buildings in a low-carbon economy driven by the transition to renewable energy and energy conservation. Owing to the lack of comprehensive investigations of carbon reduction potential in PFs [78, 82], future studies should adopt life cycle assessment as a regular methodology to quantitatively assess the carbon reduction potential and strategies for improvement from design, construction to operation, production, and selling, including related material and energy consumption.

### PF products strengthening the resilience of urban food supply chain

The PFs are effective and stable approaches to achieving high-yield and high-quality horticultural production. By shortening distribution chains and ensuring the consistent availability of horticultural products without food safety issues, PFs enhance the resilience of urban food supply [5]. PFs should be integrated into urban food data systems to maximize their impact, considering crop cultivation, agricultural product processing, market distribution, and consumption. Such integration would enable comprehensive monitoring and management of the urban food chain. Moreover, the resilience of urban food (e.g. horticultural products from PFs) supply chains can be enhanced by AI, which increases the flexibility of food supply and improves the ability to respond to fluctuating demand [147]. AI facilitates efficient transportation, warehousing, and distribution planning, thereby optimizing resource allocation and profitability [148]. Consequently, adopting AI-based methods such as AIoT, deep learning, and big data analytics, could support the advanced automation of PFs and enable intelligent decision-making, reducing food miles and offering both environmental and economic benefits.

## Conclusion

In conclusion, PFs are advanced agricultural systems enabling the multilayer cultivation of horticultural plants in a fully controlled environment, providing a promising solution for year-round, high-yield, and high-quality production in urban areas. The review highlights current and potential benefits of PFs from economic perspectives (e.g. selection of high-profitability plant species and application of cutting-edge technologies for precise environmental control) and environmental perspectives (e.g. transitioning to clean energy, increasing energy use efficiency, reusing output waste, reducing food transport distances, and integrating intelligent control). Moreover, this review proposes further innovations and applications that could contribute to addressing challenges because of high energy use, construction, and operation costs. The integration of emerging technologies to enhance the economic viability and environmental sustainability of PFs represents a comprehensive and promising strategy for advancing urban horticulture, which could strengthen the resilience of urban food systems and contribute to fundamental research and industrial applications.

## Data Availability

All data supporting the findings of this review are available within the article.
